# The Functional, Social and Economic Impact of Acute Encephalitis Syndrome in Nepal – a Longitudinal Follow-Up Study

**DOI:** 10.1371/journal.pntd.0002383

**Published:** 2013-09-12

**Authors:** Michael J. Griffiths, Jennifer V. Lemon, Ajit Rayamajhi, Prakash Poudel, Pramina Shrestha, Vijay Srivastav, Rachel Kneen, Antonieta Medina-Lara, Rupa R. Singh, Tom Solomon

**Affiliations:** 1 Brain Infections Group, Department of Clinical Infection, Microbiology and Immunology, Institute of Infection and Global Health, University of Liverpool, Liverpool, United Kingdom; 2 Department of Paediatric Neurology, Alder Hey Children's National Health Service Foundation Trust, Liverpool, United Kingdom; 3 Department of Paediatrics, Kanti Children's Hospital, Kathmandu, Nepal; 4 Department of Paediatrics, National Academy of Medical Sciences, Kathmandu, Nepal; 5 Department of Paediatrics, B.P. Koirala Institute of Health Sciences, Dharan, Nepal; 6 Health Economics Group, Peninsula College of Medicine and Dentistry, University of Exeter, Exeter, United Kingdom; 7 Department of Neurology, The Walton Centre National Health Service Foundation Trust, Liverpool, United Kingdom; 8 National Consortium for Zoonosis Research, University of Liverpool, Leahurst Campus, Wirral, United Kingdom; University of Washington, United States of America

## Abstract

**Background:**

Over 133,000 children present to hospitals with Acute Encephalitis Syndrome (AES) annually in Asia. Japanese encephalitis (JE) accounts for approximately one-quarter of cases; in most cases no pathogen is identified and management is supportive. Although JE is known to result in neurological impairment, few studies have examined the wider impact of JE and AES on patients and their families.

**Methodology/Principal Findings:**

Children (aged 1 month–14 years) with AES were assessed 5–12 months after discharge from two Nepali hospitals. Assessment included clinical examination, the Liverpool Outcome Score (LOS) - a validated assessment of function following encephalitis, questionnaires about the child's social participation since discharge, and out-of-pocket costs to the family. Children were classified as JE or ‘other AES’ based on anti-JE virus antibody titres during acute illness. Contact was made with the families of 76% (73/96) of AES children. Six children had died and one declined participation. 48% (32/66) reported functional impairment at follow-up, most frequently affecting behaviour, language or limb use. Impairment was more frequent in JE compared to ‘other AES’ cases (68% [13/19] versus 40% [19/47]; p = 0.06). 49% (26/53) had improvement in LOS between discharge and follow-up. The median out-of-pocket cost to families, including medical bills, medication and lost earnings was US$ 1151 (10 times their median monthly income) for children with severe/moderate impairment and $524 (4.6 times their income) for those with mild/no impairment (P = 0.007). Acute admission accounted for 74% of costs. Social participation was limited in 21% of children (n = 14).

**Conclusions/Significance:**

Prolonged functional impairment was common following AES. Economic impact to families was substantial. Encouragingly, almost half the children improved after discharge and most reported sustained social participation. This study highlights a need for long-term medical support following AES. Rationalisation of initial expensive hospital treatments may be warranted, especially since only supportive treatment is available.

## Author Summary

More than 133,000 children present annually to hospitals in Asia with clinical features of acute brain infection (Acute Encephalitis Syndrome [AES]). Japanese encephalitis accounts for one-quarter of cases in Asia. With no specific treatments for AES, management is largely supportive. AES commonly causes neurological problems. However, few studies have examined long-term outcome or the economic cost of AES. We followed up 72 Nepali children 5–12 months after acute hospital admission for AES and studied their neurological function, social participation and the out-of-pocket costs to the family. At follow-up, 6 children had died and 48% of survivors had impaired function. Behaviour, language or limb impairments were common. Encouragingly, almost half the children reported improved function at follow-up compared to hospital discharge. The economic impact was substantial; $1151 US dollars (10 times their monthly income) among families with children suffering severe/moderate impairment. Acute admission represented 74% of total costs. Few families reported limitations in their child's social participation. The functional problems experienced by these children highlights their need for long-term medical support. The substantial economic costs to families suggest rationalisation of acute care costs may be warranted. Families reported their child maintaining social participation, implying a positive attitude to social engagement.

## Introduction

In Asia more than 133,000 children suffer Acute Encephalitis Syndrome (AES) annually [1]. The most commonly identified cause of AES in Nepal and Asia is Japanese encephalitis (JE) virus which accounts for around one quarter to one third of cases [2], [3]. Both JE and AES exhibit a higher case frequency among children compared with adults [1], [2], . More than 20,000 children are thought to die from JE annually, and many more suffer neurological impairment [4]. A variety of infectious agents and immune mediated processes, such as acute demyelinating encephalomyelitis (ADEM), can cause encephalitis [6]. Recent studies in Vietnam [7], Papua New Guinea [8] and England [9] found no specific cause for 59%, 63% and 37% of paediatric cases respectively, despite comprehensive diagnostic testing.

Studies of outcome among JE patients report widely varying results, with death in 4–30% and long term neurological impairment in 22–94% [10]–[16]. For children with AES in Asia, death is reported in 4–29% of cases and functional impairment in 2.9–25% [3], [8], [10], [17]–[19]; however few studies of AES have examined outcome beyond 3 months or provided detail regarding the types of neurological impairment experienced. This is surprising given more children have AES of unknown cause than have JE.

Our previous work identified a large spectrum of behavioural disturbances and impaired school performance following JE [11]. However, no studies have specifically assessed limitations in a child's ability to participate in school, social and everyday life activities following AES.

Children with disabilities can have limited participation in everyday life and social activities. Participation is affected by motor and cognitive function, communication skills, age, gender and environmental factors [20]. Reduced participation has been shown to have a negative impact on quality of life in disabled children [21]. Improving inclusion and participation for people with disabilities is the ultimate aim of the World Health Organization's ‘Community Based Rehabilitation’ programme [22]. Although there are scores for assessing participation of children with disability in high income countries [20], [21], [23], [24], as far as we are aware, there is no such score for use in a resource poor setting.

Healthcare in Nepal, as in many low income countries, is not free. Patients are usually billed for medical care in both government and private hospitals. To date, there have been no studies which have looked at the costs of AES in a low resource country. A few studies have evaluated the economic cost of JE. Studies have estimated costs associated with JE through information from hospital administrators or neurologists, as well as by extrapolation of costs from other infectious conditions [25]–[27]. One study in Cambodia also ascertained ‘out of pocket’ costs to the family, by interviewing carers of children with JE up to 90 days after discharge from hospital [28].

JE is a vaccination priority in Nepal. In 2009 the JE vaccine was introduced into the child routine immunization programme in 16 districts in Nepal where JE is endemic (there are 75 districts in total) [29]. Despite this, there are currently no data regarding the economic burden of JE or AES. Establishing these costs is an important step towards assessing the cost effectiveness of management options for AES in Nepali children.

The aim of this study is to provide an overview of the functional impairment, social participation and economic costs experienced by Nepali children and their families following JE and other AES illness.

## Methods

### Ethics statement

Ethical approval for the study was granted by the Institutional Review Committee of Kanti Children's Hospital, the Nepal Health Research Council and the Ethics Review board of the Liverpool School of Tropical Medicine. Written informed consent was obtained from the parents or guardians of all children who participated in the study.

### Setting and recruitment

Children with suspected encephalitis were recruited from two public hospitals in Nepal. The hospital sites were: Kanti Childrens' Hospital (KCH), Kathmandu, the only government-run children's hospital in Nepal and BP Korala Institute of Health Sciences (BPKIHS), a large autonomous institution with a busy paediatric department in Dharan, Eastern Nepal. Children aged 1 month–14 years, who fulfilled the clinical and laboratory criteria for Acute Encephalitis Syndrome, based on the World Health Organization definition (below), were recruited into the study. Participants were assessed prior to hospital discharge, and were then followed-up once 5–12 months later, during April–July 2011.

The following criteria, based on the WHO definition [30], were used:

- Acute Encephalitis Syndrome (AES): Acute fever (less than 14 days duration) with altered mental state and/or new onset seizures (excluding simple febrile convulsions) with no positive identification of non-viral pathogens (e.g. bacteria or parasites) in the CSF or blood.
- AES - Confirmed JE: AES with IgM antibodies (≥40 units) to JEV in a single (CSF and/or serum) sample as detected by IgM-capture Enzyme linked immunosorbent assay (ELISA).
- ‘other AES’: AES where the above criteria were not met
  - ‘other AES - JE negative’: AES with an absence of anti-JEV IgM antibodies based on a negative test for a sample collected ≥day 10 of illness onset.
  - ‘other AES - JE status unknown’: AES where there was no testing for anti-JEV IgM antibodies or samples were taken too early in the illness to exclude JE.

### Assessment procedures

Assessment at hospital discharge involved a clinical examination of the child and interview with the family to complete the Liverpool Outcome Score questionnaire (described below). Clinical review involved a neurological examination which included assessment of coma status using the modified Glasgow coma scale for infants and children.

One follow-up assessment was undertaken for each child, 5–12 months after hospital discharge (median 8 months). Assessment at follow up involved a clinical examination of the child and three questionnaires with the family or carers (described below). Carers were contacted by telephone and invited to re-attend the hospital with their children. Families who agreed to participate, but did not wish to attend the hospital, were invited to complete the assessment over the telephone (economic costs and findings from clinical examination were not obtained for these participants). The assessments were performed by local clinical research officers trained in the use and delivery of the questionnaires.

1. *Liverpool Outcome score (LOS)*; The LOS is a simple validated tool for assessing functional impairment in children following acute encephalitis [31]. The LOS includes questions about the functional impairment of the child, comparing their child's function to community peers of the same age [31]. The child's outcome classification is defined by their lowest score achieved for any of the 15 domains assessed. A score of 5, 4, 3 or 2, corresponds to an outcome classification of no, mild, moderate or severe functional impairment respectively. Death was scored as 1. Each child was also assigned a ‘mean LOS’ by adding together the scores for each question and dividing by the number of questions asked. Mean score ranged from 2–5. A lower score indicates more severe functional impairment.
2. *Child and adolescent scale of participation (CASP)*; the impact of AES on the child's participation in everyday life and social activities was assessed using a questionnaire based on the CASP. The original CASP, composed of 20 questions, asks about participation in the home, the community and in school. It has been validated and used among American children with acquired brain injury [23], [32]. The content of the CASP was modified for use in this study following consultation with local Nepali paediatricians, other health care workers, and parents. This modification involved the exclusion of questions which overlapped with the LOS or were agreed to be culturally inappropriate (Questionnaire S1). The remaining questions (n = 10) asked about social participation at home (n = 4), participation in the community (n = 3) and participation at school (n = 3). Parents were asked to compare their child to community peers of the same age. A question was regarded as age appropriate if the parents/guardians reported that community peers of the same age as their child participated in the activity (e.g. attended school). Each question was scored from 5-2: 5 - age expected participation, 4 - somewhat limited participation, 3 - very limited participation and 2 - unable to participate. Questions regarded as age inappropriate were not scored. The mean CASP score was calculated by adding together the scores for each question and dividing by the number of age appropriate questions asked. Mean score ranged from 2–5. A lower score indicates more limited social participation.
3. *Economic questionnaire*; The costs of AES paid by each child's family, termed out-of pocket costs, was assessed using an economic questionnaire produced in collaboration with local paediatricians, other health care workers, and parents (Supplementary File S2). The first section of the questionnaire consisted of a short household survey based on content from the Nepal Living Standards Survey 2 [33]. This provided baseline information on parents' level of education and household income. Level of education was scored 1–9 corresponding to: 1- no formal schooling, 2- incomplete primary, 3- complete primary, 4- incomplete secondary, 5-complete secondary education and 6–9 different levels of higher education (Questionnaire S2). Total monthly household income was calculated by adding income in wages and any income earned ‘in kind’, i.e. paid in goods, or food produced for personal consumption. The second section of the questionnaire included questions about out-of-pocket costs to the family secondary to the child's illness. Acute costs included the hospital bills for the child's original admission, such as hospital bed, medication and investigations. Acute non-medical costs included transport to hospital, accommodation and food for the family during the acute admission. Income lost by the carers due to missing work while their child was admitted was also estimated. Post-discharge costs included purchase of medication and medical aids, and transport costs to purchase medication or attend appointments with medical doctors or traditional healers. The total cost of AES illness was calculated by adding acute admission and post-discharge costs for each participant. Values were recorded in Nepalese rupees (NPR), the local currency, but converted into United States dollars (US$) using the exchange rate for 2011 from the International Monetary Fund (1 US$ = 78.3 NPR).
4. *Clinical Examination*; When children attended the follow-up assessment at the hospital, they underwent a full general and neurological examination. This information was used to confirm the history and findings from the LOS.

### Statistical analysis

Data was entered in to Microsoft Excel and descriptive analyses performed. For statistical tests of difference and correlations, analysis was undertaken using SPSS version 18. For binary data, Fisher's exact test was used. For categorical and non-parametric data, the Mann Whitney U test was used. Agreement between mean LOS and CASP scores among the follow-up participants were measured by initially generating a Bland Altman plot and then computing the limits of agreement [34]. These limits were defined as the mean difference between the scores ±2 standard deviation of the difference. The proportion of scores that fell within the limits of agreement was calculated. For all data, a two sided test at the 95% confidence level was used to define statistical significance. No assumptions were made about the outcomes of participants who were lost to follow-up.

## Results

### Participant baseline characteristics

**Figure 1:**
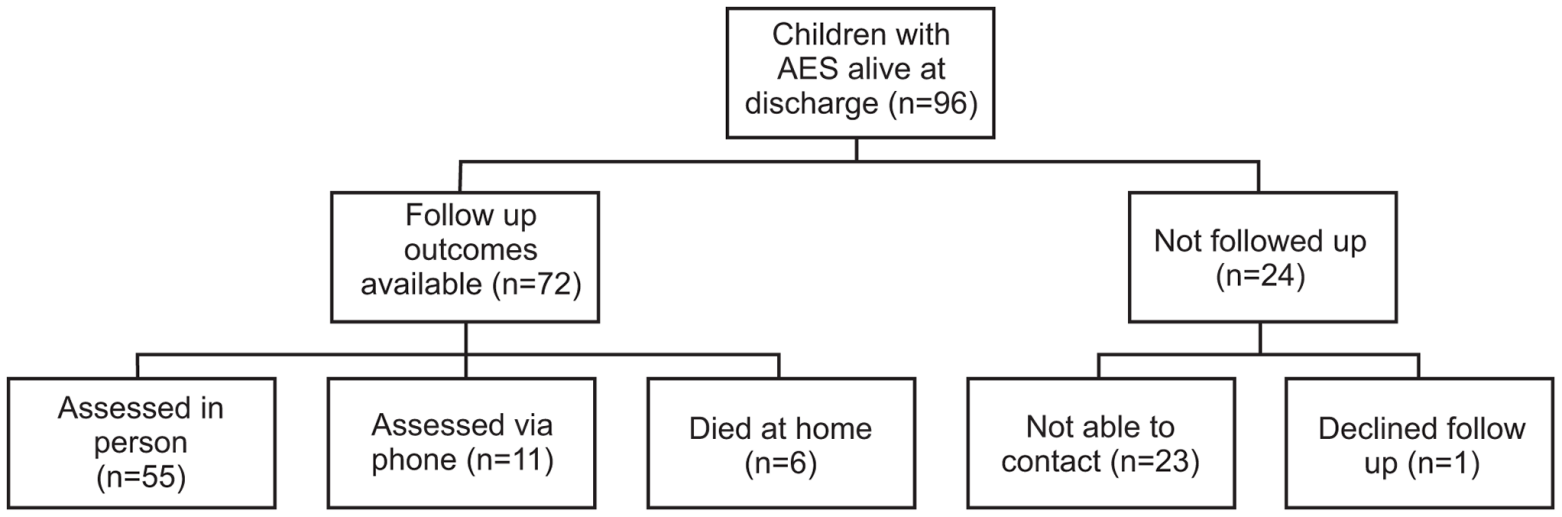
Flow diagram of study participants' recruitment and follow-up. All children admitted to hospital fitting WHO criteria for AES who were alive at discharge were attempted to be followed-up (n = 96). Seventy-two families were successfully contacted. Among these families, six children had died and 1 declined to participate further. The remaining 66 children participated in follow-up.

Ninety-six children with AES, including 34 with confirmed JE, were discharged alive, of whom 55 were followed up in person and 11 by telephone interview (Table 1). Six subjects died prior to follow-up, 23 families could not be contacted and 1 declined participation (Fig. 1). The median age for the 72 children with whom contact was made was significantly higher than for the 24 that were not (9 [range: 1–14] versus 6 [1–14] years; p = 0.029). There were no other significant differences between groups (Table 1). There was no difference in the median follow-up interval between those with confirmed JE and ‘other AES’ (8 [range: 7–12] versus 8 [6–10] months; p = 0.63) or those with severe or moderate (LOS 2/3) versus mild or no functional (LOS 4/5) impairment (8 [range: 5–10] versus 8 [5–12]; p = 0.14). Participants came from four of the five health regions and 31 of the 75 health districts across Nepal.

**Table 1:** Baseline characteristics for all patients with Acute Encephalitis Syndrome eligible for study inclusion.

	All eligible AES patients	Followed up	Not Followed up
No. patients in each group	96	72	24
Confirmed JE	31 (32.9)	22 (30.6)	9 (37.5)
Other AES	65 (67.7)	50 (69.4)	15 (62.5)
Male	58 (60.4)	45 (62.5)	13 (54.2)
Age, years	7.5 (1–14)	9 (1–14)	6 (1–14)*
Discharge GCS‡	15 (3–15)	15 (3–15)	15 (3–15)
Discharge LOS¶	3 (2–5)	3 (2–5)	3 (2–5)

Characteristics presented as number (%) or median (range).

AES, Acute Encephalitis Syndrome; No., number; GCS, modified Glasgow coma scale (score 3–15); LOS, Liverpool Outcome Score (1 [died] – 5 [no impairment]); na, not applicable.

* Significant difference in the median age among patients Followed and Not Followed up, p = 0.029.

‡ Discharge GCS was not available for 3 patients who were followed up.

¶ Discharge LOS was not available for 25 patients (19 followed up and 6 not).

### Functional impairment following AES

Thirty-two (48%) of the 66 children followed-up had functional impairment (LOS 2–4). Impairment was more common in JE than ‘other AES’ cases (68% [13/19] versus 40% [19/47]; p = 0.06). Among the ‘other AES’ cases, there were no significant differences between the ‘other AES-JE negative’ and ‘other AES-JE status unknown’ groups in the proportion of patients that died after discharge or exhibited functional impairment (Table 2).

**Table 2:** Outcome for all Acute Encephalitis Syndrome patients included in the study.

	All AES patients	Confirmed JE	Other AES	Subgroups of ‘Other AES’		P value
				JE negative	JE status unknown	
Total no. of patients in each group	72	22	50	31	19	-
Died post discharge (LOS 1)	6 (8.3)	3 (13.6)	3 (6.0)	2 (6.4)	1 (5.3)	0.36
Alive at follow-up	66	19	47	29	18	-
Any impairment at follow-up (LOS 2, 3, 4)	32 (48.5)	13 (68.4)	19 (40.4)	13 (44.8)	6 (33.3)	0.06
Severe impairment (LOS 2)	7 (10.6)	4 (21.0)	3 (6.4)	3 (10.3)	0 (0)	0.10
Moderate impairment (LOS 3)	9 (13.6)	4 (21.0)	5 (10.6)	3 (10.3)	2 (11.1)	0.27
Mild impairment (LOS 4)	16 (24.2)	5 (26.3)	11 (23.4)	7 (24.1)	4 (22.2)	1.00
No Impairment (LOS 5)	34 (51.5)	6 (31.6)	28 (59.6)	16 (55.2)	12 (66.7)	0.06

Clinical outcome for all Acute Encephalitis Syndrome patients included in the study. Outcomes presented as number in each group with proportion (%). Where outcome involved death, the denominator was based on total number of families contacted. Otherwise denominator was based on the number of patients alive at follow-up in the group. P values correspond to differences between ‘Confirmed JE’ and ‘Other AES’ groups across categories of impairment. Significance calculated using Fisher's exact test.

AES, Acute Encephalitis Syndrome; No., number; LOS, Liverpool Outcome Score (1 [died] – 5 [no impairment]).

### Types of neurological impairment following AES

**Figure 2:**
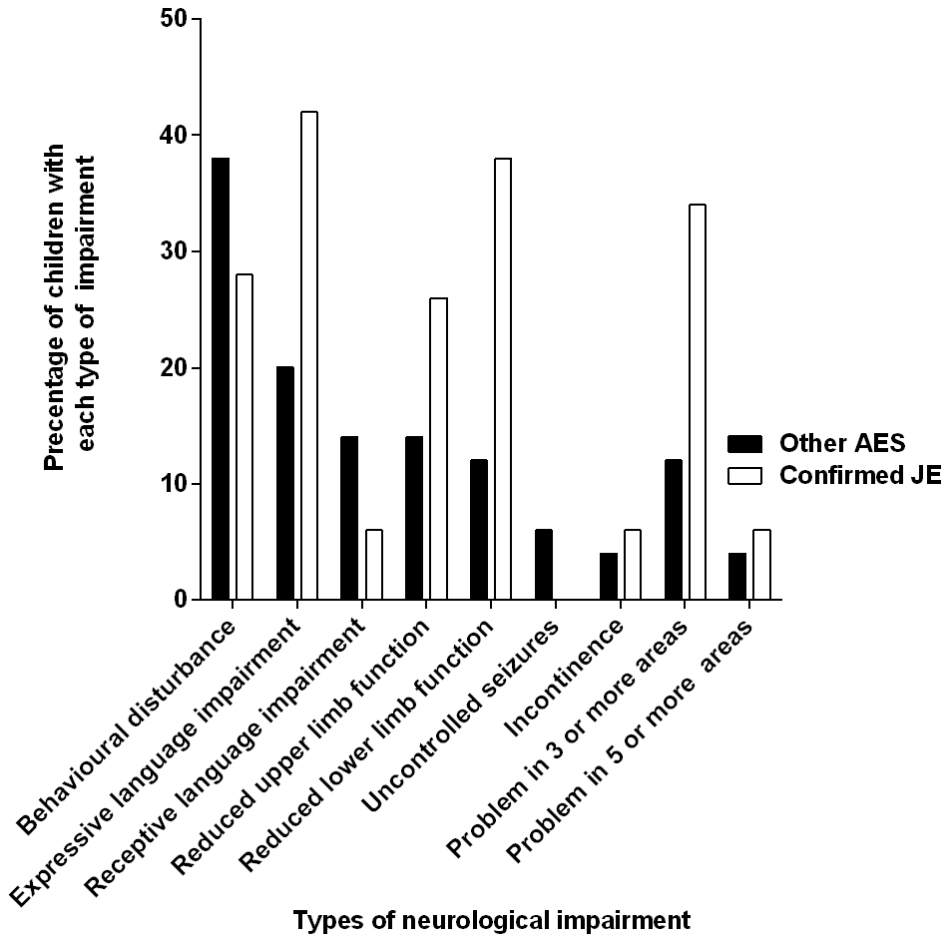
Comparison of neurological impairment experienced by children at follow-up classified with JE or ‘Other AES’. Graph displays proportions (%) of the different types of neurological impairment experienced by children that were alive at follow-up after hospitalisation with AES (n = 66). Children were classified as suffering from JE or ‘Other AES’ based on their anti-JE virus antibody titres measured during acute admission (see Methods). Neurological impairment was identified by reviewing clinician based on the history. White bars; JE patients (n = 19). Black bars; ‘other AES’ patients (n = 47).

The most frequently reported problems following AES were behavioural disturbance, language problems, including both expressive and hearing/receptive language difficulties, and reduced limb function (Fig. 2). Although neurological sequelae were more common in those with confirmed JE, the types of impairment were similar in children with confirmed JE and other AES.

### Recovery profile following AES

Twenty-six (49%) of 53 participants had a better LOS at follow-up than discharge, with 9 (17%) moving from a classification of severe functional impairment (LOS 2) to moderate functional impairment or better (LOS 3–5) and of these, 5 (10%) making a full recovery (LOS 5). Seventeen (32%) children had the same LOS at discharge and follow-up. Nine (17%) participants had a worse LOS at follow-up, of whom 3 had died. Three additional children, for whom no discharge LOS was available, died during the follow-up period. Overall, 12 (21%) of 56 subjects had a worse LOS since discharge.

**Figure 3:**
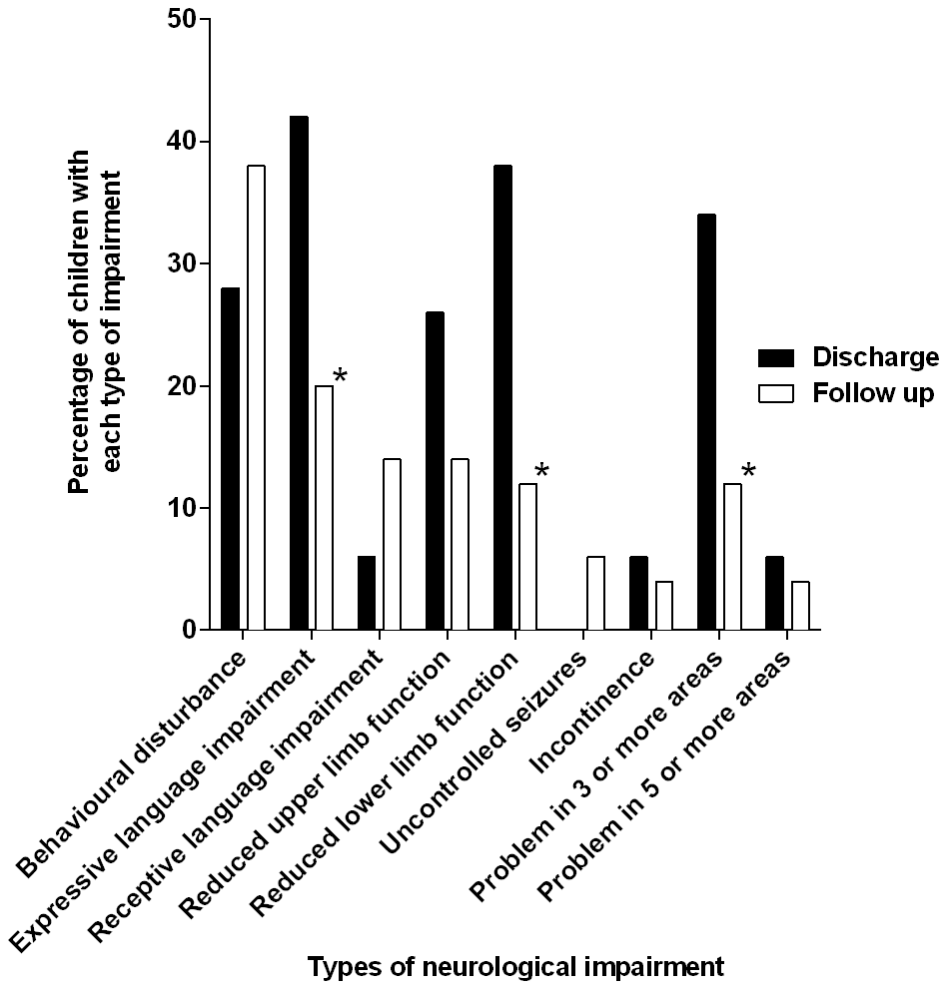
Comparison of neurological impairment experienced by children with AES at discharge and at follow-up. Graph displays proportions (%) of the different types of neurological impairment experienced by children alive at discharge and at follow-up who had a Liverpool Outcome Score measured at both time-points (n = 50). Neurological impairment was identified by reviewing clinician based on the history. White bars; Problem at follow-up. Black bars; Problem at discharge. * Significant difference (P<0.05) in the proportion of children who exhibited a specific type of neurological impairment at discharge and at follow-up. Significance was measured by Fisher's exact test.

Most neurological impairments were less common at follow-up compared to hospital discharge. Impairments of expressive speech (10/50 [20%] vs. 21/50 [42%]; p = 0.03) or lower limb function (6/50 [12%] vs. 19/50 [35%]; p = 0.005) were significantly less frequently reported at follow-up compared to discharge. However, behavioural disturbance, hearing/receptive language problems, and uncontrolled seizures were reported more frequently at follow-up than at discharge (Fig. 3). Use of healthcare services was not specifically investigated during this study, but two families reported involvement of a physiotherapist to assist with their child's motor problems.

### Economic impact of AES

For the 54 families who completed the economic questionnaire; 41 (79%) of 52 fathers and 22 (41%) of 54 mothers reported ever receiving any primary school education. Most families (40/54 [74%]) reported residing in a rural area. The occupation most frequently reported was farming.

The median monthly household income, which included income in the form of goods received or produced for personal consumption, was US$ 115 (range: 11–575). With a median household size of 6 individuals, the annual estimated income per family member was US$ 230.

The median total out-of-pocket cost of AES to the family (which included costs associated with acute hospital admission and up to 12 months after discharge) was US$ 618 (range: 140–3959), which was 5.4 times the median monthly family income. The median total cost of acute hospital admission, including transportation, hospitals bills, carers accommodation, food and loss of earnings was US$ 473 (range: 52–3831), which was 4 times the median monthly income (Table 3). For the 15 carers who gave a breakdown of hospital bills, medication was the largest itemised expense, with a median cost of US$ 243 (range: 27–1277) per child; representing 57% of their admission cost.

**Table 3:** Educational and economic data among the 54 families interviewed, grouped by Liverpool Outcome Score.

	All	LOS 2 or 3	LOS 4 or 5
Number of participants in each group	54	15	39
Proportion of fathers who ever attended school	41/52 (79)	10/15 (67)	31/37 (84)
Proportion of mothers who ever attended school	22/54 (41)	5/15 (33)	17/39 (44)
Median number of household members	6 (2–16)	6 (4–16)	5 (2–16)
Median household income, US$	115 (11–575)	115 (11–511)	112 (35–575)
Median total cost of admission, US$	473 (52–3831)	754 (179–3831)*	447 (52–1277)
Median cost of medication, US$†	243 (27–1277)	268 (102–1277)	236 (27–702)
Median admission length, days	9 (2–34)	11 (3–34)	9 (2–31)
Proportion sold an asset for admission costs	8/53 (15)	2/15 (13)	6/38 (16)
Proportion borrowed money for admission costs	46/53 (87)	12/15 (80)	34/38 (89)
Proportion missed work during acute illness	44/54 (81)	13/15 (87)	31/39 (79)
Median work days missed during acute illness	15 (4–45)	16 (7–45)	15 (4–35)
Median earnings lost during acute illness, US$	38 (0–192)	38 (0–153)	38 (0–192)
Proportion who spent money after discharge	40/54 (74)	11/15 (73)	29/39 (74)
Median spent after discharge, US$	32 (0–3004)	51 (0–3004)	31 (0–294)
Median spent on medication after discharge, US$¶	32 (1–255)	51 (5–255)	32 (1–255)
Proportion who missed work following child's discharge	19/54 (35)	10/15 (67)§	9/39 (23)
Median work days missed after discharge	30 (4–250)	60 (7–250)	23 (4–60)
Median earnings lost after discharge (of those who missed work, US$	51 (0–460)	51 (0–460)	45 (0–319)
Median total costs (admission and after discharge), US$	618 (140–3959)	1151 (254–3959)‡	524 (140–1679)

Results presented as number in each group with proportion (%) or Median (range) grouped by Liverpool outcome score (LOS) at follow-up; LOS 2 or 3 represents Severe or Moderate impairment; LOS 4 or 5 represents Mild or No Impairment; US$, United Sates Dollars.

*§‡ Significant difference in median values or proportion of patients between ‘LOS 2 or 3’ and ‘LOS 4 or 5’ groups (p = 0.048*, p = 0.004^§^ and p = 0.007^‡^ respectively). Significance measured via Mann Whitney U or Fisher's Exact Test.

† 15 participants provided specific information on admission medication cost.

¶ 29 participants provided information on discharge medication costs.

All families reported direct costs associated with the acute hospital admission. The majority of families (46/53 [87%]) had to borrow money to cover the cost of acute admission and 8/53 (15%) had to sell an asset. Most carers (44/54 [81%]) missed work during their child's acute illness, with a median of 15 days (range: 4–45) missed and US$ 38 (range: 0–192) of income lost. Some carers (8/54 [15%]) missed work but did not lose income; some reasons included being granted paid leave, or that they had finished cultivation on their farm.

The costs after discharge were much lower than during acute admission. Forty of 54 (74%) participants reported out-of-pocket costs following their child's discharge. Their median expenditure was US$ 32 (range: 0–3004).

Purchase of medication was the most frequent out-of-pocket expenditure, reported by 29 (60%) of 48 families (median cost US$ 32 [range: 1–255]). Nineteen (35%) of 54 families reported taking time off work after the child's discharge. The median estimated income lost among these families was US$ 51 (range: 0–460). The median number of days missed was 30 (range: 4–250).

### Influence of functional impairment on economic impact

The total median cost of AES was significantly higher for children with severe or moderate functional impairment compared to children with mild or no functional impairment at follow-up (US$ 1151 [range: 254–3959] versus 524 [range: 140–1679]; p = 0.007). These total out-of-pocket costs corresponded to 10 and 4.6 times the median monthly income of the participants' families. The former group also had higher admission costs (US$ 754 [range: 179–3831] versus 447 [52–1277]; p = 0.048) and costs after discharge (US$ 51 [0–3004] versus 31 [0–294]; p = 0.28).

A higher proportion of carers for children with severe/moderate functional impairment took time off work after discharge compared to carers for children with mild/no functional impairment (10/15 [67%] versus 9/39 [23%]; p = 0.004). The carers of children with severe/moderate functional impairment also took longer median time off work (median 60 [range: 7–250] versus 23 [4–60] days; not significant) and lost more income (median US$ 51 [range: 0–460] versus 45 [0–319]; not significant). Details are provided in Table 3.

Among all participants, four (two with severe/moderate impairment and two with mild/no impairment) reported unusually high post-discharge costs with one child taken to India for treatment, at a cost of US$ 3003, one transferred to another hospital at a cost of US$ 255 and two taken to a traditional healer at median cost of US$ 179. Removing these cases, carers for children with severe/moderate functional impairment still reported significantly higher total out-of-pocket costs (p = 0.025). There were no significant differences in household income or education levels of the parents between children with severe/moderate functional impairment compared to those with mild/no impairment following AES (Table 3).

### Participation in everyday activities following AES

Overall, 14 (21%) of the 66 carers who completed the CASP reported limitations in their child's participation either within the home, the community or the school environment. Five (8%) of sixty six children had limitations in all three environments. Among school aged children, 10 (16%) of the 62 subjects reported limitations with participation at school, of which 7 had stopped attending school. Among school aged children with moderate or severe impairments, 7 (50%) of the 14 children were still attending school full time. Reasons for reduced school participation included seizures, mobility, behavioural and financial problems.

We anticipated that children who exhibited limited functional impairment (relatively high LOS) would also exhibit limited impairment in their social participation (relatively high CASP score). Where children had both assessments undertaken at follow-up, we compared the mean CASP scores against their corresponding mean LOS to assess their levels of agreement.

**Figure 4:**
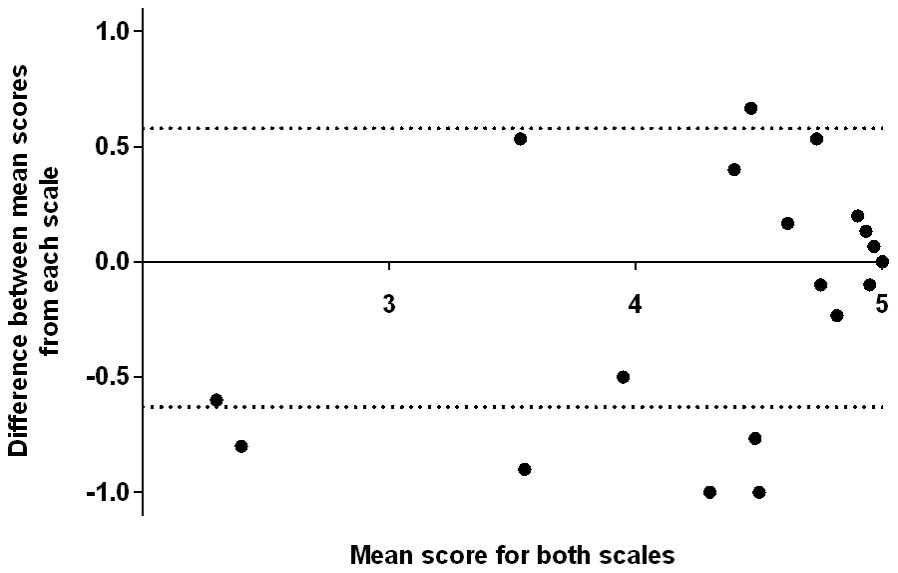
Assessment of agreement between LOS and CASP scores at follow-up among AES children. Bland-Altman plot displays mean (x-axis) and difference (y-axis) in the mean LOS and CASP score among the children with AES (n = 66) who had both scores assessed at follow-up. Solid line indicates mean difference between the two scores. Dotted lines indicate the limits of agreement (±2 standard deviations away from the mean difference). Sixty children (91%) had scores for both the CASP and LOS that were within the limits of agreement. Six children had significantly different scoring for the CASP and LOS. Five children, plotting below the lower limit of agreement, exhibited a relatively low CASP score compared to their LOS. One child, plotting above the upper limit of agreement, exhibited a relatively high CASP score compared to their LOS. Reviewing the individual scores for the former 5 children identified that they experienced other factors beyond functional impairments recognised via the LOS that limited their social participation (Results).

Overall, the mean CASP score demonstrated reasonable agreement against the mean LOS. Sixty (91%) of 66 children assessed by both scores at follow-up exhibited mean CASP scores and LOS that fell within the limits of agreement during Bland-Altman analysis (Figure 4). Among the six participants that fell outside these limits, five families reported their child having lower social participation (based on the CASP) than may be expected for their child's reported functional ability (based on the LOS). Reviewing the parental responses for these children; two had behavioural problems that limited social participation; one child had visual impairment; one child no longer attended school due to the family's worse financial circumstances since the AES illness; and one child no longer attended school due to moderate mobility impairment since the AES illness, compounded by the family living relatively far from the school.

One family reported their child having higher social participation than may be expected for their child's functional ability. The child was reported to have developmental delay prior to AES illness.

## Discussion

This is the first study to examine the functional, economic and social impact of AES in parallel following hospital discharge. Although this study was undertaken in Nepal, AES is prevalent in Asia and so we would expect the findings to be of relevance throughout the area [1]. We show a substantial impact of AES on a child's function several months after their acute illness. There were considerable out-of-pocket costs of AES illness to families, with the median cost being more than five times their estimated monthly income. The predominant costs were linked with the acute hospital admission. Total costs were significantly higher for children with severe or moderate functional impairment compared to those with mild or no impairment. Encouragingly, among participants in whom LOS was measured at discharge and follow-up, 49% reported improved function up to 12 months later. Despite the high levels of functional impairment, few families reported limitations in their child's social participation.

This study highlights the high burden of disability following AES illness of undetermined cause. Almost one-tenth of children died following discharge; around half of the survivors exhibited functional impairment up to 12 months after hospital discharge. Proportionally, impairment was more frequent among JE confirmed AES cases compared to ‘other AES’ cases, as seen in previous studies [3], [10], [19]. This finding highlights the value of JE vaccination programmes. However, in terms of absolute numbers more children suffered functional impairment in the ‘other AES' group’. The types of impairment reported in the JE and ‘other AES’ groups were similar. In line with previous JE studies, behavioural difficulties were most frequently reported at follow-up [11], [35]. Language or limb movement difficulties were next most frequently reported.

Function improved at follow-up in almost half of the participants; a fifth exhibited a worse outcome; and a third exhibited the same outcome. These findings for AES, are in line with the results from our previous study of long-term outcome for JE among Malaysian children [11]. Although the use of health services was not a specific focus of this study, few families reported involvement with health professionals after discharge. Input from psychologists, speech and language therapists, physiotherapists and occupational therapists may be useful to children with AES when locally available. The changes in patients' levels of impairment following hospital discharge, emphasises the clinical need for follow-up of AES patients. The results also provide useful information for health care professionals when discussing the prognosis of AES with Nepali patients.

The most striking finding of this study was the considerable out-of-pocket costs of AES illness to families, being more than 5 times their estimated monthly income. These costs rose to 10 times the monthly income for families of children with moderate or severe functional impairment. The estimated annual household income per family member was US$ 230, considerably lower than the US$ 490 estimated Gross National Income per capita for Nepal reported by the World Bank for 2010 [36]. Typical of many health problems, families with relatively low income may be disproportionately affected by AES illness in Nepal. In contrast, school attendance rates among the parents were comparable to the national rates reported in 2011, suggesting families of low educational attendance were not disproportionately admitted to hospital with AES [37].

This study has provided the first description of the out-of-pocket costs experienced by families following AES. Given cases of AES are more common than those with confirmed JE [1], describing the costs linked to AES is especially important. Acute hospital admission was the major cost, representing three-quarters of the total out-of-pocket cost. Acute admission was also identified as the major cost associated with JE illness in a Cambodian study that interviewed affected families [28]. Among the 15 Nepali families who were able to give a breakdown of admission costs, medication was the main expense, representing over half the acute admission cost. Similarly, medication was identified to be the predominant acute medical cost associated with JE illness in Indonesia [25].

This study is unique, in that functional assessment and out-of-pocket costs were collected alongside one another. This allowed us to demonstrate, that children with severe/moderate functional impairment reported higher admission costs than those with mild/no impairment. These costs are probably due to the children being more severely unwell and having a longer admission. Although attempts to rationalise acute hospital treatment should be balanced against the needs of patients and medical staff to explore all reasonable avenues of treatment, a more detailed analysis of acute costs may help determine where hospital treatment could be streamlined. Acute bacterial meningitis can have overlapping clinical features with AES. Herpes simplex virus encephalitis is also an infrequent cause of AES in Asia [7]. Beyond these exceptions, there is no specific drug treatment for patients presenting with AES. Enhancing diagnostic exclusion of these conditions may help rationalise the use of medication among patients hospitalised with AES. In turn, this could significantly reduce the economic impact to families. The relatively low post-discharge out-of-pocket costs to families in Nepal are in line with previous findings from Cambodia [28]. These low costs may reflect limited access to services for children following encephalitis.

Over three-quarters of families reported maintenance of their child's social participation following AES; the majority (84%) of school age children, including half of the children with moderate or severe functional impairments continued to attend full-time education. A similar proportion of children attended school in Cambodia and Vietnam following JE [35]. A slightly lower proportion of school attendance (75%) was reported among Malaysian children following JE [11]. The latter may reflect the longer period of follow-up (up to 8 years) undertaken in the study or a different attitude to social participation among the population.

Overall, the results of the CASP questionnaire showed good agreement with the LOS. The good agreement between these outcome scales implies that children with high functional ability (high LOS) following AES illness also tend to have a high level of social participation (high CASP). However, several children (n = 5) had a low level of social participation despite a high LOS. These children suffered behavioural disturbance, visual impairment or changes in their external environment (such as family financial difficulties) since the AES illness that limited their social participation. The findings demonstrate that the CASP score can identify children at risk of poor social participation that would otherwise not be detected based solely on their level of functional impairment. To our knowledge, there are no other publications comparing these two scales.

On the whole, Nepali families appeared to have a positive attitude towards social engagement of their child within the community. More detailed research on the families' attitudes to social participation is now warranted. With further work, a modified CASP score could be a useful tool for assessing social participation in children with disabilities in a low resource setting.

There were several limitations to this study. The main reason for loss of follow-up was inability to contact the family by phone. Follow-up may have been improved by linking with community health care teams to contact families directly. There was a higher median age in those who were followed up than those who were not. As only one family declined assessment, we are unable to provide a reason for this finding. We assumed the carers' responses to the questionnaires were valid. We did not collect additional indicators (e.g. responses from the child or school teacher) to authenticate these reports. Carers recalled out-of-pocket costs from the hospital admission several months previous. More accurate costs and detail about what specific medical expenses were incurred may have been obtained by gathering information during the child's acute admission.

In conclusion, although attention has been focussed on JE, AES causes considerable prolonged functional impairment to children, with substantial out-of-pocket costs to the family. Consideration towards the cost of in-patient treatment may reduce the economic impact to families, and release funds for hospital follow-up, community services and rehabilitation following AES. Further research involving local health professionals and carers would identify which services would be most useful to AES patients long-term and help to determine how best to support community health services for these children.

## Supporting Information

Checklist S1 STROBE checklist.
(DOC)

Questionnaire S1 Modified Child and Adolescent Scale of Participation (CASP) questionnaire.
(DOC)

Questionnaire S2 Economic questionnaire.
(DOC)
