# Supplementary material for: The Functional, Social and Economic Impact of Acute Encephalitis Syndrome in Nepal – a Longitudinal Follow-Up Study
Source: PLoS Negl Trop Dis. 2013 Sep 12;7(9):e2383. doi: 10.1371/journal.pntd.0002383 (PMC3772013; doi:10.1371/journal.pntd.0002383)
Supplement: Checklist S1 — STROBE checklist. (DOC) [file pntd.0002383.s001.doc]

STROBE Statement—Checklist of items that should be included in reports of ***cohort studies***

|  | Item No | Recommendation |
| --- | --- | --- |
| **Title and abstract** | 1 | (*a*) Indicate the study’s design with a commonly used term in the title or the abstract *- in title (lines 1-2)* |
|  |  | (*b*) Provide in the abstract an informative and balanced summary of what was done and what was found *- done (lines 31-56)* |
| Introduction | | |
| Background/rationale | 2 | Explain the scientific background and rationale for the investigation being reported  *– done (lines 81-120)* |
| Objectives | 3 | State specific objectives, including any prespecified hypotheses *–aim of study documented (lines 121-123)* |
| Methods | | |
| Study design | 4 | Present key elements of study design early in the paper *– done* |
| Setting | 5 | Describe the setting, locations, and relevant dates, including periods of recruitment, exposure, follow-up, and data collection *– methods (lines 132-139)* |
| Participants | 6 | (*a*) Give the eligibility criteria, and the sources and methods of selection of participants (lines 132-152) . Describe methods of follow-up  *methods (158-165)* |
|  |  | (*b*) For matched studies, give matching criteria and number of exposed and unexposed *– not applicable* |
| Variables | 7 | Clearly define all outcomes, exposures, predictors, potential confounders, and effect modifiers. Give diagnostic criteria, if applicable -*; functional impairment of participant, social participation of participant and out-of pocket costs to family - all defined in methods (lines 167-220)* |
| Data sources/ measurement | 8* | For each variable of interest, give sources of data and details of methods of assessment (measurement). Describe comparability of assessment methods if there is more than one group  *How assessment of outcomes was undertaken and the questionnaires used (via Liverpool Outcome Score (LOS); modified Child and Adolescent Scale of Participation (CASP); and economic questionnaire ) are described in Methods and or Supplementary Information.*  *Each patient’s scores from LOS and CASP were compared using Bland-Altman plot as described in Methods (lines 228-233)* |
| Bias | 9 | Describe any efforts to address potential sources of bias. *How scoring was handled for children who were too young to undertake all activities / tasks described in the questionnaires were explained in methods (lines 193-196)* |
| Study size | 10 | Explain how the study size was arrived at *described in Flowchart (Figure 1)* |
| Quantitative variables | 11 | Explain how quantitative variables were handled in the analyses. If applicable, describe which groupings were chosen and why *How quantitative variables were handled is described (lines 225-234)* |
| Statistical methods | 12 | (*a*) Describe all statistical methods, including those used to control for confounding – *statistical methods described (lines 225-234)* |
|  |  | (*b*) Describe any methods used to examine subgroups and interactions *– no subgroup analyses undertaken* |
|  |  | (*c*) Explain how missing data were addressed  *No assumptions /adjustments were made for participants lost to follow-up* – *(line 233)* |
|  |  | (*d*) If applicable, explain how loss to follow-up was addressed *- Not applicable* |
|  |  | (*e*) Describe any sensitivity analyses *–Not applicable* |
| Results | | |
| Participants | 13* | (a) Report numbers of individuals at each stage of study—eg numbers potentially eligible, examined for eligibility, confirmed eligible, included in the study, completing follow-up, and analysed *Flow chart provided– Figure 1* |
|  |  | (b) Give reasons for non-participation at each stage *Provided in results* |
|  |  | (c) Consider use of a flow diagram –*Figure 1* |
| Descriptive data | 14* | (a) Give characteristics of study participants (eg demographic, clinical, social) and information on exposures and potential confounders – *described in results (lines 238-247) and Table 2* |
|  |  | (b) Indicate number of participants with missing data for each variable of interest –*number of participants with data for each variable of interest documented in results and / or table legends* |
|  |  | (c) Summarise follow-up time (eg, average and total amount) *median and range presented (lines 138-139)* |
| Outcome data | 15* | Report numbers of outcome events or summary measures over time *in results / tables* |
| Main results | 16 | (*a*) Give unadjusted estimates and, if applicable, confounder-adjusted estimates and their precision (eg, 95% confidence interval). Make clear which confounders were adjusted for and why they were included – *raw unadjusted results presented* |
|  |  | (*b*) Report category boundaries when continuous variables were categorized *– median and range presented in results and / or tables* |
|  |  | (*c*) If relevant, consider translating estimates of relative risk into absolute risk for a meaningful time period *– not relevant* |
| Other analyses | 17 | Report other analyses done—eg analyses of subgroups and interactions, and sensitivity analyses – *not applicable* |
| Discussion | | |
| Key results | 18 | Summarise key results with reference to study objectives *– done (lines 363-71)* |
| Limitations | 19 | Discuss limitations of the study, taking into account sources of potential bias or imprecision. Discuss both direction and magnitude of any potential bias *– discussion (lines 454-463)* |
| Interpretation | 20 | Give a cautious overall interpretation of results considering objectives, limitations, multiplicity of analyses, results from similar studies, and other relevant evidence *–*  *study findings interpreted in context of study limitations and previous published results* |
| Generalisability | 21 | Discuss the generalisability (external validity) of the study results *– study findings placed in context of previous published results in discussion* |
| Other information | | |
| Funding | 22 | Give the source of funding and the role of the funders for the present study and, if applicable, for the original study on which the present article is based *–documented in manuscript submission form* |

*Give information separately for exposed and unexposed groups.

**Note:** An Explanation and Elaboration article discusses each checklist item and gives methodological background and published examples of transparent reporting. The STROBE checklist is best used in conjunction with this article (freely available on the Web sites of PLoS Medicine at http://www.plosmedicine.org/, Annals of Internal Medicine at http://www.annals.org/, and Epidemiology at http://www.epidem.com/). Information on the STROBE Initiative is available at http://www.strobe-statement.org.
