# Supplementary material for: The Functional, Social and Economic Impact of Acute Encephalitis Syndrome in Nepal – a Longitudinal Follow-Up Study
Source: PLoS Negl Trop Dis. 2013 Sep 12;7(9):e2383. doi: 10.1371/journal.pntd.0002383 (PMC3772013; doi:10.1371/journal.pntd.0002383)
Supplement: Questionnaire S1 — Modified Child and Adolescent Scale of Participation (CASP) questionnaire. (DOC) [file pntd.0002383.s002.doc]

**Full 20 item content from the Child and Adolescent Scale of Participation (CASP) (Bedell, 2009)**

CASP responses

4: Age expected: Your child participates in the activities the same as or more than other children his or her age (with or without assistive devices or equipment).

3: Somewhat limited: Your child participates in the activities somewhat less than other children his or her age (may also need occasional supervision or assistance).

2: Very limited: Your child participates in the activities much less than other children his or her age (may also need a lot of supervision or assistance).

1: Unable: Your child can not participate in the activities, although other children his or her age do participate.

0: Not applicable: Other children your child’s age would not be expected to participate in the activities.

Home participation

1. Social, play, or leisure activities with family members at home (e.g., games, hobbies, “hanging out”)

2. Social, play or leisure activities with friends at home (can include conversations on the phone or internet)

3. Family chores, responsibilities and decisions at home (For younger children this may be getting things or putting things away when asked, or helping with small parts of household chores; for older children this may be more involvement in household chores and decisions about family activities and plans)

4. Self-care activities (e.g., eating, dressing, bathing, combing or brushing hair, using the toilet)

5. Moving about in and around the home

6. Communicating with other children and adults at home

Neighbourhood and community participation

7. Social, play, or leisure activities with friends in the neighbourhood and community (e.g., casual games, “hanging out”, going to public places such as a movie theatre, park or restaurant)

8. Structured events and activities in the neighbourhood and community (e.g., team sports, clubs, holiday or religious events, concerts, parades and fairs)

9. Moving around the neighbourhood and community (e.g., public buildings, parks, restaurants, movies) – please consider your child’s primary way of moving around, NOT his or her use of transportation

10. Communicating with other children and adults in the neighbourhood and community

School participation

11. Educational (academic) activities with other children in the classroom

12. Social, play, and recreational activities with other children at school (e.g., “hanging out”, sports, clubs, hobbies, creative arts, lunchtime or recess activities)

13. Moving around at school (e.g., getting to and using the bathroom, playground, cafeteria, library, or other rooms and services that are available to other children his or her age)

14. Using educational materials and equipment available to other children in his or her classroom, or using materials and equipment that have been modiﬁed for your child

15. Communicating with other children and adults at school

Home and community living activities

16. Household activities (e.g., preparing some meals, doing laundry, washing dishes)

17. Shopping and managing money (e.g., shopping at stores, ﬁguring out correct change)

18. Managing daily schedule (e.g., doing and completing daily activities on time; organizing and

adjusting time and schedule when needed)

19. Using transportation to get around in the community (e.g., to and from school, work, social

or leisure activities). Driving a vehicle or using public transportation are both applicable.

20. Work activities and responsibilities (e.g., task completion, punctuality, attendance, and getting

along with supervisors and co-workers)

**Simplified 10 item CASP questionnaire used in this study**

| **CASP responses** (each question is scored out of 5 points)  5: Age expected: Your child participates in the activities the same as or more than other children his or her age (with or without assistive devices or equipment).  4: Somewhat limited: Your child participates in the activities somewhat less than other children his or her age (may also need occasional supervision or assistance).  3: Very limited: Your child participates in the activities much less than other children his or her age (may also need a lot of supervision or assistance).  2: Unable: Your child can not participate in the activities, although other children his or her age do participate.  0: Not applicable: Other children your child’s age would not be expected to participate in the activities (Question will be excluded from score). | | |
| --- | --- | --- |
| **Home participation** | |  |
| 1. Social, play, or leisure activities with family members at home (e.g., playing games) | |  |
|  | |  |
| 2. Family chores, responsibilities, and decisions at home (For younger children this may be getting things or putting things away when asked, or helping with small parts of household chores; for older children this may be more involvement in household chores and decisions about family activities and plans) | |  |
|  |
|  |
|  | |  |
| 3. Self-care activities (e.g., eating, dressing, washing, toileting) | |  |
|  | |  |
| 4. Moving about in and around the home | |  |
|  | |  |
| **Neighborhood and community participation** | |  |
| 5. Socialising and playing with friends and other children in the village/ community | |  |
|  | |  |
| 6. Participation in structured events /activities in the community (e.g, festivals and religious events) | |  |
|  |
| 7. Moving around the village/ community (please consider your child’s primary way of moving around, NOT his or her use of transportation) | |  |
|  |
|  | |  |
| **School participation (the yes/no questions are not scored)** | |  |
| Was your child attending school before the hospital admission? Please circle yes or no | yes | no |
|  |  |  |
| Is your child currently at school? Please circle yes or no (If yes please skip to question 8) | yes | no |
|  |  |  |
| If they are not at school, is this because of problems they have had since they were in hospital (For example: Cant get to school due to mobility, behaviour problems) | yes | no |
| If they are not at school please give the reason  If they are at school, please comment on their participation in the following school activities | | |
| 8. Educational (academic activities with other children in the classroom) | |  |
|  | |  |
| 9. Social, play, and recreational activities with other children at school | |  |
|  | |  |
| 10. Moving around at school (e.g., getting around the classroom, outdoor play area, or other rooms) | |  |
|  |
| Are there any other activities that your child cannot join in with that they were able to do before they were in hospital? | | |
