# Supplementary material for: The Functional, Social and Economic Impact of Acute Encephalitis Syndrome in Nepal – a Longitudinal Follow-Up Study
Source: PLoS Negl Trop Dis. 2013 Sep 12;7(9):e2383. doi: 10.1371/journal.pntd.0002383 (PMC3772013; doi:10.1371/journal.pntd.0002383)
Supplement: Questionnaire S2 — Economic questionnaire. (DOC) [file pntd.0002383.s003.doc]

### **ECONOMIC QUESTIONNAIRE**

| Sex 1. M  2. F | Age | **Relation to child** (write ‘self’ if it is the child) | **Education**(see codes page 3) | Occupation (see codes page 3) | **IN THE LAST MONTH** | | | |  |
| --- | --- | --- | --- | --- | --- | --- | --- | --- | --- |
|  |  |  |  |  | **In paid job**  1. Yes  2. No | **Working days** | **Income per month** | **In kind paid job**  1. Yes  2. No | **What if paid in in kind (eg wheat, vegetables)** |
|  |  |  |  |  |  |  |  |  |  |
|  |  |  |  |  |  |  |  |  |  |
|  |  |  |  |  |  |  |  |  |  |
|  |  |  |  |  |  |  |  |  |  |
|  |  |  |  |  |  |  |  |  |  |
|  |  |  |  |  |  |  |  |  |  |
|  |  |  |  |  |  |  |  |  |  |
|  |  |  |  |  |  |  |  |  |  |
|  |  |  |  |  |  |  |  |  |  |

# Date: ID: Residence: 1 Rural 2 Urban 3 Peri-Urban District

VDC Total no. of people living in the household:

DD/MM/YY

l

# MEMBER OF HOUSEHOLD CHARACTERISTICS: Please include all household members including the participant and any people working abroad (table continued on page 4 if more room is needed)

1. Main roofing material of the house you now live in?

1 Grass thatched

2 Tin with no ceiling

3 Corrugated tin roof with

unfinished ceiling

4 Corrugated tin roof with finished

ceiling

5 Tile with unfinished ceiling

6 Tile with finished ceiling

77 Other (specify)

2. Does your house have any of the following?

N Y DA

Electricity 1 2 99

A television 1 2 99

Kitchen stove 1 2 99

A refrigerator 1 2 99

A radio 1 2 99

A bicycle 1 2 99

An ox cart 1 2 99

77 Other (specify)

HOUSEHOLD CHARACTERISTICS

1. Main roofing material of the house you now live in?

1 Thatch

2 Corrugate

3 Tiles

77 Other (specify)

2. Type of walls of the house you now live in?

1 Burned brick

2 Unburned brick

3 Mud

77 Other (specify)

3. Is there a toilet (flush to septic tank) in the house?

1 Yes

2 No

4. Do you have access to clean water?

1 Yes

2 No

8. Who of the people that live in the house you are living in contribute to the household income?

1 Father

2 Mother

3 Sibling(s)

4 Uncle(s)/aunt(s)

77 Other (specify)

9. Approximately how much money did your family receive as income (in rupees) last month?

10. Approximately how much money did your family receive as income (in kind) last month. Give amount in rupees?

10. Approximately how much food did your family produce last month for you own consumption (if family are farmers). Give value in rupees

5. Type and number of livestock you have now?

Number

1 Cattle

2 Chickens

3 Pigs

4 Goats

5 Sheep

77 Other (specify)

6a. Do you own land for cultivation?

1 Yes

2 No

6b. If yes, how many ropanies?

7. Does your household have any of the following?

N Y

Electricity 2 1

A radio 2 1

A television 2 1

A refrigerator 2 1

A bicycle 2 1

A mobile 2 1

A car 2 1

**Number**

# Child Fever Episodes

20a. If yes, how much did you pay for medications (rupees)?

88 Not applicable

20b. If yes, how much did you pay for investigations eg CT scan (rupees)?

88 Not applicable

21. If yes, how much did you pay per hospital day (rupees)?

88 Not applicable

22. Did you have to pay for accommodation for you or the carer?

1 Yes

2 No Go to question 24

23. If yes, how much did you pay for accommodation? (per day)

24. How much did you pay for meals? (per day)

Total cost of hospitalisation (if parts unknown)

25. Did you have to do any of the following in order to meet the cost of hospitalisation and other expenses?

Y N What/Amount

Sell any asset 1 2

Borrow money 1 2

Gift or donation 1 2

Sell your labour 1 2

Credit treatment 1 2

77 Other

CARER CHARACTERISTICS

26. Who took care of the child during the hospitalisation?

relation to the child

age

M F

sex 1 2

88 Not applicable

11. Approximately how much money did your family spend last month (in rupees)?

12. Who is the main carer for your child?

1 Mother

2 Father

3 Sibling

77 Other (specify)

HOSPITALISATION COSTS (for the AES admission)

14. How many days in hospital (with AES-*don’t ask parents use acute study documents)?*

15. What mode of transport did you use for going to the hospital?

1 Walking

2 By bicycle

3 By bus

4 By taxi

5 Private car

6 Motorbike

77 Other (specify)

16. Did you spend anything on transport for going to the hospital?

1 Yes

2 No Go to question 16

17. If yes, how much did you spend on transport?

18. How long did the trip last? (how long was the journey?)

88 Not applicable

19. Did you have to pay for the hospitalisation?

1 Yes

2 No Go to question 22

27. Did the carer miss studies or work (either paid or unpaid) as a result of the child’s hospitalisation?

1 Yes

2 No

88 Not applicable

**28. If yes, approximately how many days did he/she miss?**

29. Did the carer lose earnings as a result of the child’s hospitalisation?

1 Yes

2 No

88 Not applicable

**30. If yes, approximately how much did he/she lose?**

**Any further comments:**

**AFTER HOSPITALISATION**

31. Who was the main carer for your child after the hospitalisation?

1 Mother

2 Father

3 Sibling

77 Other (specify)

32. Did the carer miss studies or work (either paid or unpaid) after the child’s hospitalisation?

1 Yes

2 No go to question 34

**33. If yes, approximately how many days did he/she miss?**

34. Did the carer lose earnings after the child’s hospitalisation?

1 Yes

2 No go to question 34

# Child Fever Episodes

# Child Fever Episodes

**34. If yes, approximately how much did he/she lose?**

35. Did the carer change to part-time work after the child’s hospitalisation?

1 Yes

2 No go to question 38

36. If yes, how many days per month did the carer work before the child’s hospitalisation?

37. If yes, how many days per month does the carer work after the child’s hospitalisation?

38. Did any other member of the family change his/her working status to become a carer as a result of the child’s hospitalisation?

1 Yes

2 No go to question 42

39. If yes, please state the relation to the child

40. If yes, how many days did the carer used to work before the child’s hospitalisation?

41. If yes, how many days does the carer work after the child’s hospitalisation?

42. Did any other member of the family change his/her studying status to become a carer as a result of the child’s hospitalisation?

1 Yes

2 No go to question 44

43. If yes, please state the relation to the child

44. Were there any additional costs after the child’s hospitalisation?

1 Yes

2 No

45. If yes, which of the following

Y N Amount

Medications 1 2

Transport 1 2

Child equipment 1 2

Credit treatment 1 2

77 Other (specify)

Total of costs after hospitalisation (if parts unknown)

46. Did you have to do any of the following in order to meet the costs after the hospitalisation?

Y N Specify

Sell any asset 1 2

Borrow money 1 2

Gift or donation 1 2

Sell your labour 1 2

Credit treatment 1 2

77 Other

Any further comments:

Coding information

To be used to fill in the occupation and education boxes on the first page table

**Education**

1 None

2 Primary incomplete

3 Primary complete

4 Secondary incomplete

5 Secondary complete

6 Technical/skilled job training

7 University incomplete

8 University complete

9 Postgraduate studies

**Occupation**

1 Farming –home garden

2 Farming -other

3 Fishing

4 Labouring

5 Office job

6 Business

7 Housework

8 Looking after children

9 Looking after sick person

10 Bed ridden

11 student

12 None

77 Other (specify)

| Sex 1. M  2. F | Age | **Relation to child** (write ‘self’ if it is the child) | **Education**(see codes page 3) | Occupation (see codes page 3) | **IN THE LAST MONTH** | | | |  |
| --- | --- | --- | --- | --- | --- | --- | --- | --- | --- |
|  |  |  |  |  | **In paid job**  1. Yes  2. No | **Working days** | **Income per month** | **In kind paid job**  1. Yes  2. No | **What if paid in in kind (eg wheat, vegetables)** |
|  |  |  |  |  |  |  |  |  |  |
|  |  |  |  |  |  |  |  |  |  |
|  |  |  |  |  |  |  |  |  |  |
|  |  |  |  |  |  |  |  |  |  |
|  |  |  |  |  |  |  |  |  |  |
|  |  |  |  |  |  |  |  |  |  |
|  |  |  |  |  |  |  |  |  |  |
|  |  |  |  |  |  |  |  |  |  |
|  |  |  |  |  |  |  |  |  |  |

# Child Fever Episodes

# Child Fever Episodes
